# The Type III Secreted Protein BspR Regulates the Virulence Genes in *Bordetella bronchiseptica*

**DOI:** 10.1371/journal.pone.0038925

**Published:** 2012-06-11

**Authors:** Jun Kurushima, Asaomi Kuwae, Akio Abe

**Affiliations:** Laboratory of Bacterial Infection, Graduate School of Infection Control Sciences, Kitasato University, Tokyo, Japan; Universidad Nacional de La Plata, United States of America

## Abstract

*Bordetella bronchiseptica* is closely related with *B. pertussis* and *B. parapertussis*, the causative agents of whooping cough. These pathogenic species share a number of virulence genes, including the gene locus for the type III secretion system (T3SS) that delivers effector proteins. To identify unknown type III effectors in *Bordetella*, secreted proteins in the bacterial culture supernatants of wild-type *B. bronchiseptica* and an isogenic T3SS-deficient mutant were compared with iTRAQ-based, quantitative proteomic analysis method. BB1639, annotated as a hypothetical protein, was identified as a novel type III secreted protein and was designated BspR (*Bordetella* secreted protein regulator). The virulence of a BspR mutant (Δ*bspR*) in *B*. *bronchiseptica* was significantly attenuated in a mouse infection model. BspR was also highly conserved in *B*. *pertussis* and *B*. *parapertussis*, suggesting that BspR is an essential virulence factor in these three *Bordetella* species. Interestingly, the BspR-deficient strain showed hyper-secretion of T3SS-related proteins. Furthermore, T3SS-dependent host cell cytotoxicity and hemolytic activity were also enhanced in the absence of BspR. By contrast, the expression of filamentous hemagglutinin, pertactin, and adenylate cyclase toxin was completely abolished in the BspR-deficient strain. Finally, we demonstrated that BspR is involved in the iron-responsive regulation of T3SS. Thus, *Bordetella* virulence factors are coordinately but inversely controlled by BspR, which functions as a regulator in response to iron starvation.

## Introduction

*Bordetella* is a genus of gram-negative β-proteobacteria and is currently divided into 9 species with different hosts [1]. *Bordetella pertussis*, *B. parapertussis*, and *B. bronchiseptica* are highly genetically related pathogens that cause respiratory diseases in animals. *B. parapertussis* can be divided into two distinct lineages, *B. parapertussis*_HU_ and *B. parapertussis*_OV_, which infect human and sheep, respectively. [2]. *B. pertussis* and *B. parapertussis*_HU_ are strictly human-adapted species and are the etiological agents of whooping cough (pertussis) [3]. *B. bronchiseptica* is a pathogen with a broad range of hosts and causes chronic infections: kennel cough in dogs, snuffles in rabbits, and atrophic rhinitis in swine [4]. Despite differences in host tropism, these three *Bordetella* species share many virulence factors and a control system for virulence [5].

A virulence-associated two-component signal transduction system composed of BvgA and BvgS (BvgAS) serves as the master regulator for most *Bordetella* virulence factors [6]. BvgS is a transmembrane sensor kinase, and BvgA is a DNA-binding responsive activator [7]. At 37°C in standard *Bordetella* medium, BvgS is autophosphorylated and eventually transfers the phosphate group to BvgA via an intramolecular, four-step phospho-relay cascade (designated as Bvg^+^ phase) [8]. Phosphorylated BvgA is then activated and binds DNA to promote the transcription of various virulence genes including adhesins, toxins, and the components of the type III secretion system (T3SS) [9]. The BvgAS system is environmentally responsive and two other Bvg-regulated phases have been characterized [10], [11], [12]. In contrast to Bvg^+^ phase, expression of Bvg-activated genes were repressed when the bacteria grown at 37°C in medium containing high concentrations of MgSO_4_ (Bvg^-^ phase). Growth at 37°C in medium containing ‘semi-modulating’ concentrations of MgSO_4_ allows the Bvg-intermediate (Bvg^i^ phase). Thus, the BvgAS system plays a key role in the virulent phase of *Bordetella* species [13].

Many gram-negative pathogens produce a T3SS, which has a needle-like structure protruding from the bacterial outer membrane and delivers effectors into host cells, thereby altering the physiological functions of infected cells [14]. Five type III secreted proteins, BteA (also referred to as BopC), BopB, BopD, BopN, and Bsp22, have been identified in *Bordetella* [15], [16], [17]. BopB and BopD form a translocation pore complex in the host membrane that acts as a conduit for effectors [15], [17]. Bsp22 forms a filamentous structure at the tip of the needle structure and associates with the pore component BopD [16]. Two type III effectors, BteA and BopN, have been identified in *Bordetella* [18], [19], [20]. BteA is localized to lipid rafts in host cells via its N-terminal region and induces necrotic cell death in various types of mammalian cells [18], [20]. BopN is translocated into the nucleus and alters the nuclear translocation of NF-κB, resulting in the up-regulation of IL-10, an anti-inflammatory cytokine [19].

The T3SS-related gene cluster, the *bsc* locus, is highly conserved among *B. pertussis*, *B. parapertussis*, and *B. bronchiseptica* [3]. The *bsc* locus consists of 29 genes encoding type III secretion apparatus components, regulators, and secreted proteins; the gene encoding BteA is separated from the *bsc* locus [18], [20], [21]. The *btr* locus is adjacent to the *bsc* locus and encodes BtrS, BtrU, BtrW, and BtrV, which are involved in the regulation of T3SS-associated genes at the transcriptional or post-transcriptional level [22], [23]. BtrS is an extracytoplasmic function (ECF) sigma factor that is positively regulated by BvgAS and is necessary for the transcriptional activation of T3SS-associated genes including *bsc, btr*, and *bteA* [23].

To further explore the function of the *Bordetella* T3SS, we searched for unknown substrates of the T3SS in *B*. *bronchiseptica* using an iTRAQ-based comparative proteomic approach and identified a novel type III secreted protein, BspR. Here, we report that BspR is involved in the regulation of various BvgAS-regulated genes encoding the T3SS, filamentous hemagglutinin, pertactin, and adenylate cyclase toxin.

## Results

### BB1639 is Secreted by the T3SS in *B. bronchiseptica*

**Figure 1:**
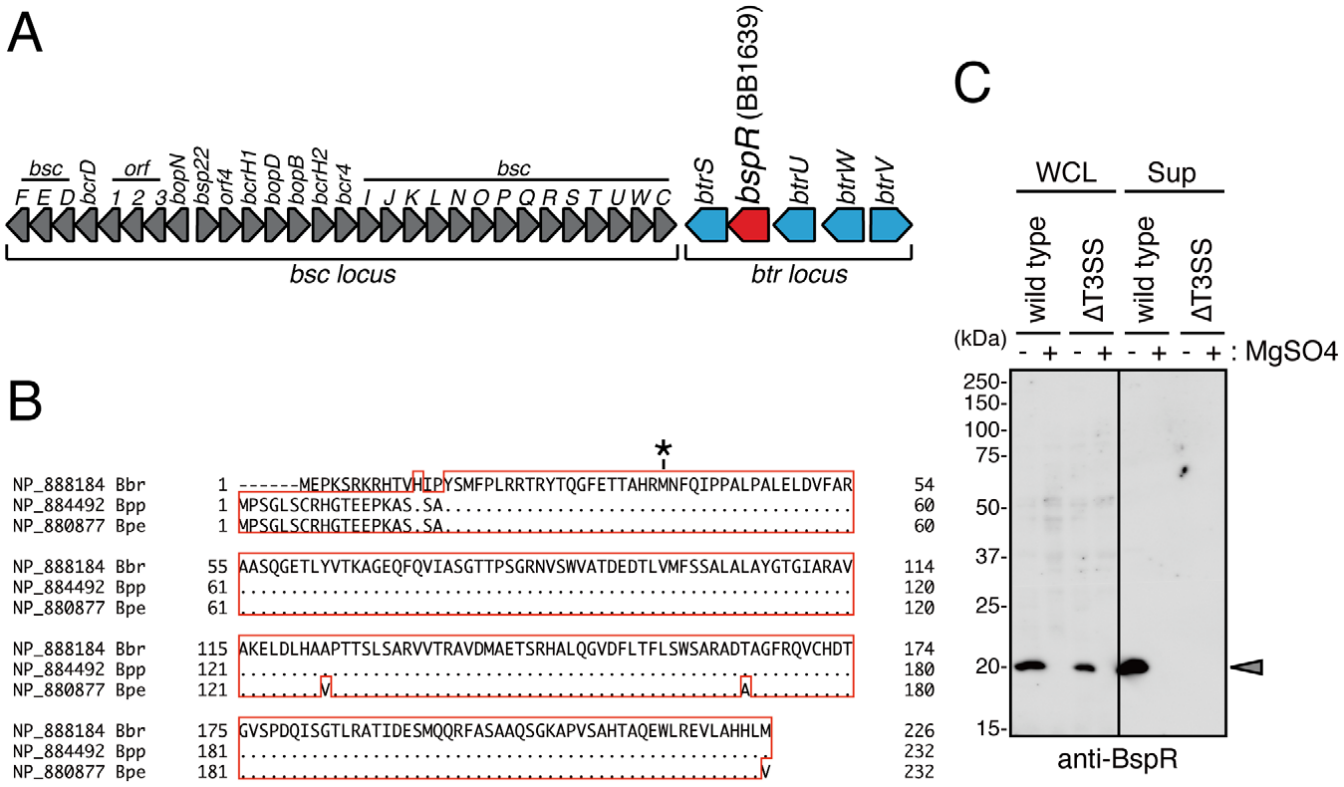
BspR is a type III secreted protein conserved in pathogenic *Bordetella* species. (**A**) Genomic organization of the *bsc* and *btr* loci in *B. bronchiseptica*. Sequence data were obtained from the *B. bronchiseptica* sequencing group at the Sanger Institute (ftp://ftp.sanger.ac.uk/pub/pathology/bb/). (**B**) BLAST alignment of BspR: BB1639 in *B*. *bronchiseptica* (Bbr), BPP2242 in *B*. *parapertussis* (Bpp), and BP2233 in *B*. *pertussis* (Bpe). The dots represent identical amino acid sequence in their respective *Bordetella* spp. The asterisk indicates the putative start methionine codon predicted by MetaGeneAnnotator (http://metagene.cb.k.u-tokyo.ac.jp/). (**C**) Immunoblot analysis using anti-BspR antibodies. Wild-type and ΔT3SS *B. bronchiseptica* were grown on SS medium in the absence or presence of MgSO_4_ under vigorous shaking at 37°C for 18 h. Whole-cell lysates (WCL) and secreted protein fraction in the supernatant (Sup) were prepared from the bacterial cultures and analyzed by immunoblot analysis using anti-BspR antibodies. The arrowhead indicates the specific signal for BspR.

To identify novel type III secreted proteins in *B. bronchiseptica*, we performed an iTRAQ-based proteomic analysis to compare the secretion profiles of a wild-type *B. bronchiseptica* strain and an isogenic mutant lacking a functional T3SS (ΔT3SS: *bscN* mutant) (Fig. 1). The total proteins recovered from each bacterial culture supernatant (Fig. S1) was labeled with iTRAQ reagents and subjected to tandem mass spectrometry (MS/MS) analysis, which determines the quantitative differences in the levels of peptides and proteins from distinct sources. The peak ratio of wild-type signal to ΔT3SS signal for each individual identified protein was calculated by analyzing the peak area of the released reporter peptide from the iTRAQ tag during MS/MS. As expected, the previously characterized type III secreted proteins BopB, BopD, BopN, BscF, Bsp22, and BteA were identified in the wild-type supernatant at a significantly higher level than in the ΔT3SS supernatant (Table 1). On the other hand, the peak ratios of T3SS-independent secreted proteins in the wild-type sample were similar to those in the ΔT3SS sample (0.72- to 1.70-fold) (Table 1). Interestingly, the peak ratio of an uncharacterized open reading frame (BB1639, NP_888184) was higher in the wild-type strain than that in the ΔT3SS strain (by 3.85-fold), suggesting that BB1639 is a candidate T3SS-dependent secreted protein. BB1639 is a hypothetical protein encoded in the *btr* locus and is located directly upstream of *btrS* (Fig. 1A). BB1639 is highly conserved in the *btr* loci of *B*. *pertussis* (BP2233, NP_880877) and *B*. *parapertussis* (BPP2242, NP_884492) (Fig. 1B), but the function of this gene in each species remains to be elucidated. In this study, we propose that the product of BB1639 be renamed BspR (*Bordetella* secreted protein regulator). We also detected BscF that is thought to be a component of the needle structure of the T3SS [3]. BscF was eliminated from further study, since the needle structures have been characterized in several T3SS-related pathogens [24].

**Table 1:** T3SS -dependent and -independent secreted proteins in *B. bronchiseptica.*

Protein	Description	Secretion	1WT:ΔT3SS	2 *p*-value
**T3SS-dependent secreted protein**				
Bsp22	type III needle tip protein	T3SS	**7.94**	<0.01
BteA	type III effector	T3SS	**7.52**	<0.01
BopB	type III translocator	T3SS	**5.88**	<0.01
BopD	type III translocator	T3SS	**5.52**	<0.01
BscF	type III needle monomer	T3SS	**5.03**	<0.01
BopN	type III effector	T3SS	**4.95**	<0.01
BB1639	uncharacterized hypothetical protein	unknown	**3.85**	<0.01
**T3SS-independent secreted protein**				
BB3110	putative autotranporter	T5SS	**1.70**	<0.01
CyaA	adenylate cyclase toxin/hemolysin	T1SS	**1.48**	<0.01
FhaS	homologue of FhaB	T5SS	**1.46**	<0.01
BapC	autotransporter	T5SS	**1.23**	<0.01
FhaL	homologue of FhaB	T5SS	**1.13**	0.01
BapB	autotransporter	T5SS	**1.11**	0.04
Prn	adhesion factor	T5SS	**1.04**	0.02
BipB	putative adhesion factor	T5SS	**0.92**	<0.01
FhaB	adhesion factor	T5SS	**0.91**	0.01
FimB	chaperone protein	T2SS	**0.72**	<0.01

1 Ratio of the amount of secreted protein of wild-type *B*. *bronchiseptica* against ΔT3SS strain.

2 Significance in the differences between wild type and ΔT3SS (t-test).

To confirm that BspR is secreted via the T3SS, we performed immunoblot analysis using anti-BspR antibodies (Fig. 1C). Intracellular BspR was detected in whole-cell lysates of both wild-type and ΔT3SS *B. bronchiseptica*. By contrast, the secretion of BspR into bacterial culture supernatants was only detected in the wild type strain and not in the ΔT3SS strain. *B*. *bronchiseptica* cultured in the presence of MgSO_4_ was shifted into the avirulent phase, which inhibited the secretion most virulence proteins including the type III secreted proteins. As expected, intracellular and secreted BspR were no longer detected in either the wild-type strain or the ΔT3SS strain in the presence of MgSO_4_. These results clearly indicate that BspR is a type III secreted protein and is expressed under the control of the BvgAS system.

### BspR Negatively Regulates the T3SS in *B*. *bronchiseptica*

**Figure 2:**
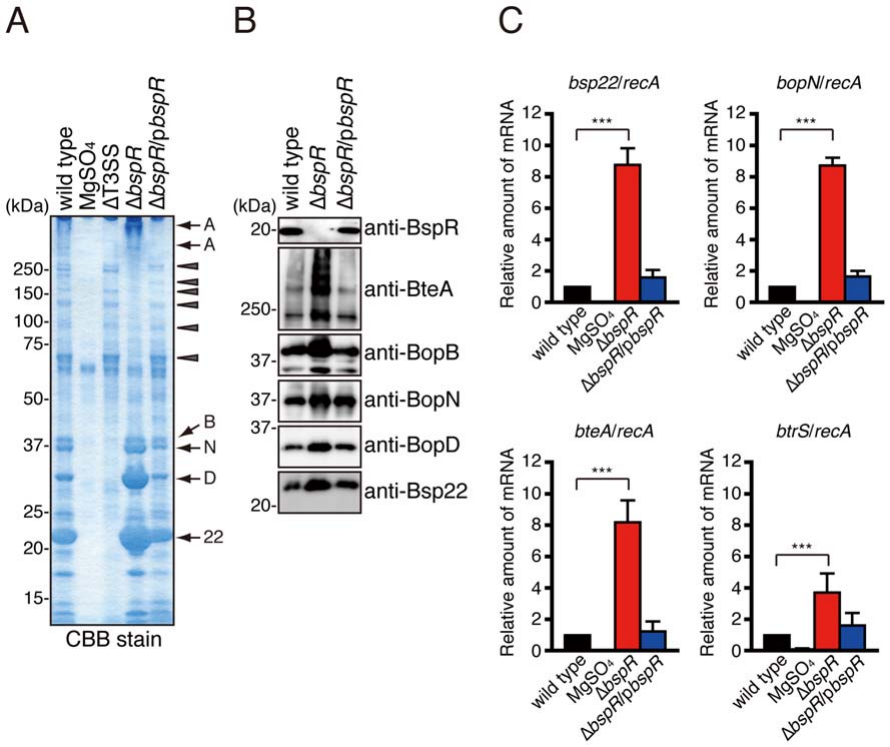
BspR negatively regulates the expression of T3SS-associated genes. *B. bronchiseptica* strains were grown in SS medium under vigorous shaking at 37°C. The wild-type strain cultured in the presence of MgSO_4_ (40 mM) was used as a negative control. (**A**) The secreted proteins isolated from bacterial culture supernatants were separated by SDS-PAGE and stained with CBB. Arrows indicate the bands corresponding to type III secreted proteins: A, BteA (multimerized); B, BopB; N, BopN; D, BopD; and 22, Bsp22. Arrowheads indicate the bands that disappeared in the absence of BspR. (**B**) Whole bacterial lysates were prepared from bacterial cultures and analyzed by immunoblotting using the following antibodies: anti-BspR, anti-BteA, anti-BopB, anti-BopN, anti-BopD, and anti-Bsp22. (**C**) Total RNA isolated from *B. bronchiseptica* strains was subjected to quantitative RT-PCR analysis. The graphs show the relative amounts of *bsp22*, *bopN*, *bteA*, and *btrS* mRNA normalized to the mRNA for the housekeeping gene *recA*. The wild-type strain cultured in the presence of MgSO_4_ (40 mM) was used as a negative control. The values represent the means ± SE from three independent experiments. ***, P<0.001.

When *B*. *bronchiseptica* was grown in liquid SS medium, a number of virulence factors, such as adhesins, toxins, and type III secreted proteins, were detected in the bacterial culture supernatants [15]. To investigate the role of BspR in protein secretion, wild-type, T3SS mutant (ΔT3SS), *bspR* mutant (Δ*bspR*), and *bspR*-complemented (Δ*bspR*/p*bspR*) *B. bronchiseptica* strains were each cultured in SS medium for 18 h. The secreted proteins were isolated from each bacterial culture supernatant and subjected to sodium dodecyl sulfate polyacrylamide gel electrophoresis (SDS-PAGE) followed by Coomassie brilliant blue (CBB) staining (Fig. 2A). Secreted protein prepared from a culture containing MgSO_4_ was used as a negative control. The type III secreted proteins BteA, BopB, BopD, BopN, and Bsp22 were detected in culture supernatant from the wild-type strain but not those from the ΔT3SS strain. Interestingly, the secretion of the type III-associated proteins was greatly increased in the Δ*bspR* strain. By contrast, the secretion profile of a complemented strain (Δ*bspR*/p*bspR*) was similar to that of the wild-type strain. To further investigate the effect of BspR on the T3SS, whole bacterial lysates were analyzed by immunoblotting (Fig. 2B). Again, the amount of type III secreted proteins (BopB, BopD, BopN, BteA, and Bsp22) in whole Δ*bspR* cells was greater than that in wild-type and Δ*bspR*/p*bspR* cells. These results suggest that BspR negatively regulates the secretion of type III secreted proteins into the bacterial culture supernatant.

To determine whether BspR regulates the expression of genes encoding type III secreted proteins at the transcriptional level, total RNA was isolated from *B*. *bronchiseptica*, and cDNA was reverse transcribed from the total RNA for qRT-PCR analysis (Fig. 2C). The relative amounts of *bsp22*, *bopN*, and *bteA* mRNA in the Δ*bspR* strain were approximately 8-fold greater than those in the wild-type strain. Interestingly, the *btrS* mRNA level in the Δ*bspR* strain was also significantly higher than that in the wild-type strain. Again, there are no significant differences in the relative amounts of mRNA between the wild-type and Δ*bspR*/p*bspR* strains. Collectively, these results indicate that BspR functions as a negative regulator of type III secreted proteins at the transcriptional level.

### A BspR-deficient Strain is Hyper-virulent in Cultured Mammalian Cells

**Figure 3:**
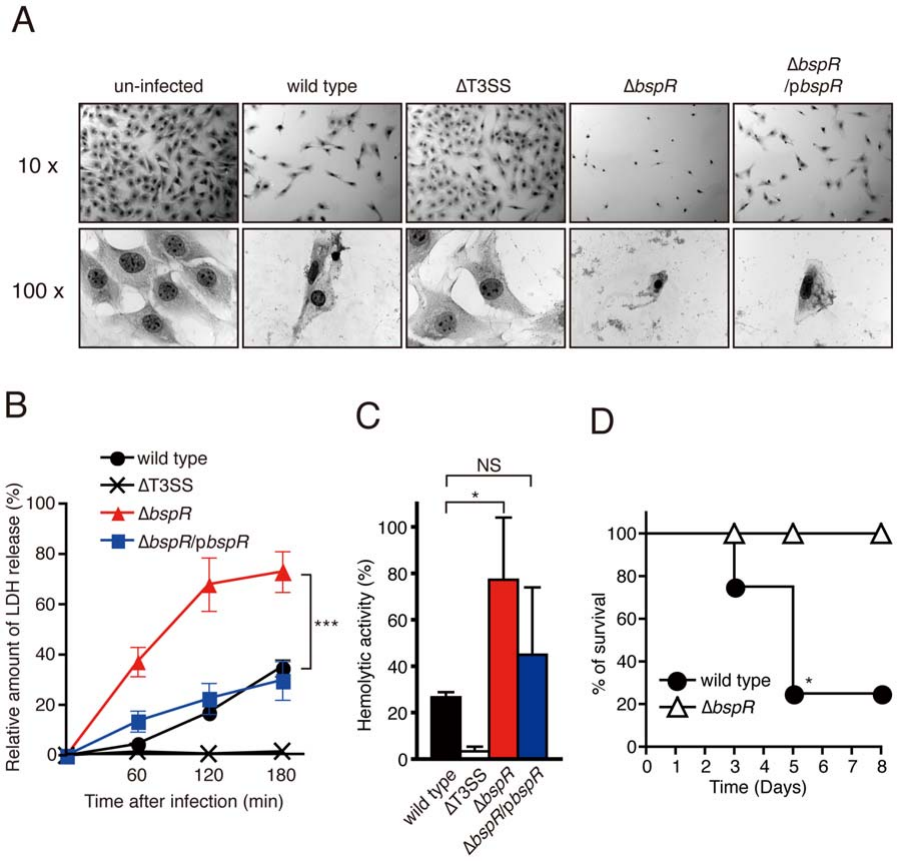
A BspR-deficient strain is hyper-virulent against cultured mammalian cells. (**A**) L2 cells were infected with the indicated *B. bronchiseptica* strains at an MOI of 20 for 20 min. Infected cells were subjected to Giemsa staining and were analyzed under a light microscope. (**B**) HeLa cells were infected with the indicated *B. bronchiseptica* strains at an MOI of 10. The amount of LDH released into the extracellular medium from infected cells was quantified at 0, 60, 120, and 180 min post-infection, and the relative cytotoxicity (%) was determined as described in the Materials and Methods section. The data for each sample are presented as the mean ± SE of three independent experiments. ***, P<0.001. (**C**) RBCs were tightly pelleted with the indicated *B. bronchiseptica* strains by centrifugation and incubated for 30 min. After incubation, the release of hemoglobin from the RBCs into the extracellular buffer was calculated by measuring the absorbance at 492 nm. The presented values are percentages of Triton X-100-lysed RBCs after subtraction of the measured lysis of untreated cells. The data for each sample are presented as the mean ± SE of three independent experiments. *, P<0.05; NS, no significance. (**D**) C57BL/6J mice were infected intranasally (5×10^6^ CFU/mouse) with a wild-type or Δ*bspR* strain. The survival of the mice (n = 4 per group) was monitored for 8 days after infection. *, P<0.05 using the log-rank test.

*B. bronchiseptica* infection induces necrotic cell death in various cultured mammalian cells, and this activity is dependent on the translocation of the BteA effector into host cells [18], [20]. To determine whether BspR affects T3SS-dependent cytotoxicity, L2 cells were infected with wild-type, ΔT3SS, Δ*bspR* or Δ*bspR*/p*bspR B. bronchiseptica* and stained with Giemsa solution to analyze cell morphology under a light microscope (Fig. 3A). Approximately 60–70% of cells infected with wild-type or Δ*bspR*/p*bspR B. bronchiseptica* were detached from the substratum, and the remainder of the adherent cells exhibited shrunken cytoplasm and condensed nuclei (Fig. 3A). The L2 cells exposed to the ΔT3SS strain showed normal morphology, similar to uninfected cells. By contrast, more than 90% of cells infected with the Δ*bspR* strain were detached, and the morphological changes observed were more severe than those in the wild-type or Δ*bspR*/p*bspR* strain. To quantify T3SS-dependent cytotoxicity, the relative amount of LDH released into the extracellular medium was measured (Fig. 3B). When the cells were infected with wild-type or Δ*bspR*/p*bspR B. bronchiseptica*, LDH release progressively increased throughout the experiment, reaching approximately 30% at 3 h after infection. By contrast, the cytotoxicity of the Δ*bspR* strain was significantly greater than that of the wild-type strain.

*B. bronchiseptica* induces the hemolysis of rabbit red blood cells (RBCs) in an adenylate cyclase toxin (CyaA)- or T3SS-dependent manner. In particular, T3SS-dependent hemolysis is caused by the formation of the pore complex containing BopB and BopD in the RBC plasma membrane, which results in membrane disruption [15], [16], [17]. In a previous report, we established a system to measure T3SS-dependent hemolytic activity [15]. To investigate whether BspR is involved in T3SS-dependent hemolysis, rabbit RBCs were exposed to *B. bronchiseptica* (Fig. 3C). The hemolytic activity of the wild-type strain was 27.8% of a Triton X-100-treated positive control. Again, the hemolytic activity of the Δ*bspR* strain was 85.5%, significantly higher than that of the wild-type strain. Taken together, the absence of BspR results in the de-repression of the T3SS, leading to enhanced T3SS-dependent phenotypes in cultured mammalian cells.

The *bspR*-deficient strain showed characteristic features including a hyper-secretion phenotype for type III secreted proteins (Fig. 3). Hyper-secretion resulted in the enhancement of T3SS-dependent cytotoxicity and hemolysis in cultured mammalian cells (Fig. 3A, B, and C), suggesting that the Δ*bspR* strain has been shifted into a hyper-virulent state. For this reason, C57BL/6J mice were infected intranasally with wild-type or Δ*bspR B*. *bronchiseptica* (Fig. 3D). Although 80% of mice infected with the wild-type strain succumbed within 5 days after infection, all mice infected with the Δ*bspR* strain survived. Thus, the virulence of the Δ*bspR* strain was clearly attenuated relative to that of the wild-type strain (Fig. 3D). The attenuation of virulence in the Δ*bspR* strain is probably attributable to dysregulation of the T3SS.

### BspR Globally Regulates Virulence Genes in *B*. *bronchiseptica*

**Figure 4:**
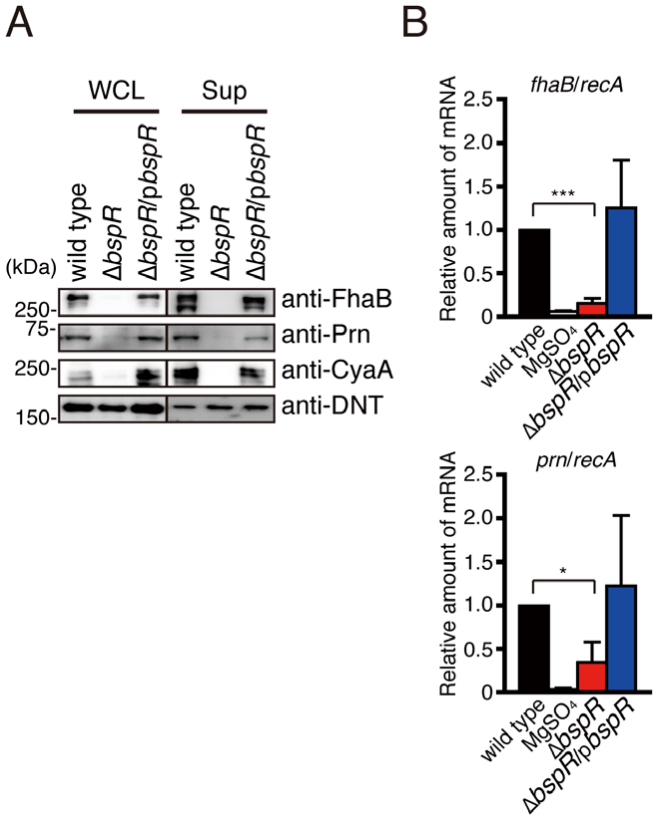
BspR affects the expression of adhesins and toxins in *B. bronchiseptica*. *B. bronchiseptica* strains were grown in SS medium under vigorous shaking at 37°C for 18 h (for immunoblotting) or 9 h (for RT-PCR analysis). The wild-type strain cultured in the presence of MgSO_4_ (40 mM) was used as a negative control. (**A**) A whole-cell lysates (WCL) and a fraction containing secreted proteins in the supernatant (Sup) were prepared from each bacterial culture and analyzed by immunoblotting using the following antibodies: anti-FhaB, anti-CyaA, anti-Prn, and anti-DNT. (**B**) Total RNA prepared from *B. bronchiseptica* strains was subjected to quantitative RT-PCR analysis. The graphs show the relative amounts of *fhaB* and *prn* mRNA normalized to the amount of mRNA for the housekeeping gene *recA*. The values are the means ± SE from three independent experiments. *, P<0.05; ***, P<0.001.

In this study, we demonstrated that the *bspR*-deficient strain shows enhanced production of type III secreted proteins. By contrast, some protein bands disappeared in the absence of BspR (Fig. 2A, arrowheads). Those bands were detected in the ΔT3SS strain but not in wild-type culture under MgSO_4_-replete conditions. These findings suggest that other T3SS-independent secreted proteins are oppositely regulated by BspR. Therefore, we decided to investigate the effect of BspR on the production of T3SS-independent virulence factors by immunoblot analysis (Fig. 4A). Filamentous hemagglutinin (FhaB), pertactin (Prn), and CyaA were detected in both whole-cell lysates and bacterial supernatants of the wild-type strain or the Δ*bspR*/p*bspR* strain. However, these bands completely disappeared in the Δ*bspR* strain. By contrast, the production and secretion of dermonecrotic toxin (DNT) were not affected by the *bspR* mutation. To further investigate whether the reduced expression of FhaB and Prn in the Δ*bspR* strain occurred at the transcriptional level, total RNA was isolated from *B*. *bronchiseptica* and analyzed by qRT-PCR to quantify the relative amounts of *fhaB* and *prn* mRNAs (Fig. 4B). *fhaB* and *prn* mRNAs were detected in wild-type *B*. *bronchiseptica* grown under MgSO_4_-depleted conditions, in which the BvgAS system is active. By contrast, the relative amount of *fhaB* or *prn* mRNA in the Δ*bspR* strain was lower than that in the wild-type and Δ*bspR*/p*bspR* strains. Collectively, BspR is involved in regulation of type III secreted proteins and other virulence factors.

### BspR Regulates Virulence Factors in Response to Iron Starvation

**Figure 5:**
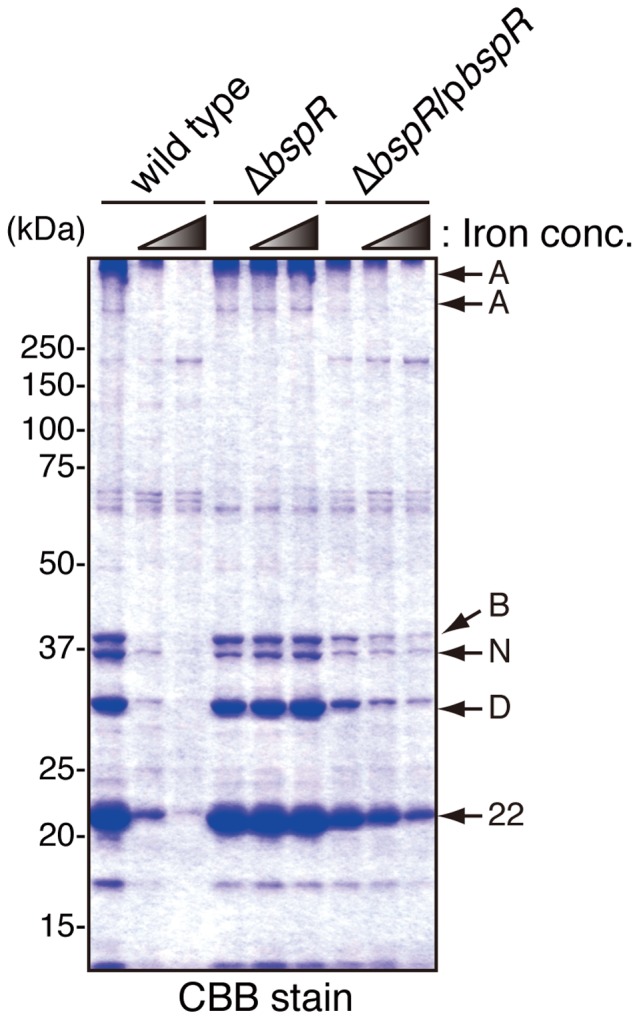
Signaling under iron starvation conditions is dysregulated in the absence of BspR. Wild-type, Δ*bspR*, and Δ*bspR*/p*bspR B. bronchiseptica* strains were grown in SS medium supplemented with iron at a final concentration of 0, 36, or 360 µM. The standard SS medium commonly used elsewhere contains iron at a concentration of 36 µM. The secreted proteins isolated from bacterial culture supernatants were separated by SDS-PAGE and stained with CBB. Arrows indicate the bands corresponding to the type III secreted proteins BteA (multimerized), BopB, BopN, BopD, and Bsp22 (A, B, N, D, and 22, respectively).

A recent study revealed that iron starvation induces the up-regulation of the T3SS [25]. Thus, the phenotypes exhibited by *B*. *bronchiseptica* under iron-starvation conditions appear to be similar to those of the *bspR*-deficient strain. To investigate the role of BspR under conditions of iron starvation, wild-type, Δ*bspR*, and Δ*bspR*/p*bspR B*. *bronchiseptica* strains were grown in SS medium supplemented with iron at a final concentration of 0, 36, or 360 µM. Then, the secreted proteins in the bacterial culture supernatants were subjected to SDS-PAGE (Fig. 5). The secretion of type III–associated proteins by the wild-type strain was fully promoted in the absence of iron and gradually decreased in response to increasing iron concentration. To our surprise, the deletion of BspR completely abolished the iron-responsive regulation of the T3SS. The hyper-secretion of type III secreted proteins by the Δ*bspR* strain was detected even with high iron concentrations. By contrast, the phenotype of the Δ*bspR*/p*bspR* complemented strain was similar to that of the wild-type strain. These results suggest that BspR regulates the expression of the T3SS in response to the iron concentration.

### BspR Functions as a Global Regulator in *B. bronchiseptica*

These findings led to our hypothesis that BspR may play a role in a global regulator. For this reason, total proteins included in whole-cell lysate from wild-type or Δ*bspR* was labeled with iTRAQ reagents and subjected to MS/MS analysis. The peak ratio of Δ*bspR* signal to wild-type signal for each individual identified protein was calculated and resulting data were presented in the rank order of the 18 most abundant proteins (Table 2) and the 18 least amounts of proteins (Table 3). The raw data are also presented in Table S1. For example, recombination-associated protein PdgC and transcriptional termination factor Rho are dramatically increased in the *bspR* mutant strain (Table 2). In contrast, biotin carboxyl carrier protein FabE and diaminobutyrate aminotransferase EctB are reduced in the *bspR* mutant strain (Table 3). These results strongly suggest that BspR functions as a global regulator in *B. bronchiseptica*.

**Table 2:** Increased proteins by the *bspR* deletion.

Protein	Description	1Δ*bspR*:WT
RdgC	recombination-associated protein	30.48
Rho	transcription termination factor	28.58
Alr	alanine racemase	26.55
FtsA	cell division protein	26.06
ParE	DNA topoisomerase IV subunit B	25.59
BB1615	hypothetical protein	21.09
BB4395	hypothetical protein	20.14
Bcr4	*bsc*-encoded protein	19.77
BB4209	putative hydrolase	19.41
EffB	electron transfer flavoprotein beta-subunit	18.88
BB3158	putative phage-related protein	18.03
PutA	trifunctional transcriptional regulator	17.54
WlbD	putative UDP-N-acetylglucosamine2-epimerase	17.22
BopN	type III effector	16.60
BvgA	transcriptional regulator	16.29
BcrH2	type III chaperone	15.56
BscJ	putative type III secreted protein	15.00
CydA	cytochrome D ubiquinol oxidase subunit I	14.59

1 Ratio of the amount of whole cell lysate proteins of Δ*bspR* against wild-type *B*. *bronchiseptica*. P-value <0.05.

**Table 3:** Decreased proteins by the *bspR* deletion.

Protein	Description	1Δ*bspR*:WT
FabE	acetyl-CoA carboxylase biotin carboxyl carrier protein	0.02
EctB	diaminobutyrate transaminase	0.03
BB2875	universal stress family protein	0.04
BB4467	putative membrane protein	0.04
BB4284	putative membrane protein	0.04
BB3926	putative cytochrome	0.05
ModB	molybdate-binding periplasmic protein precursor	0.08
BB2813	putative uncharacterized protein	0.10
AtpF	ATP synthase subunit b	0.11
BB1948	putative exported protein	0.12
MmsA	putative methylmalonate-semialdehydedehydrogenase	0.13
BipA	outermembrane protein	0.14
BB3328	cytochrome C oxidase subunit	0.15
BB3101	putative oxidoreductase	0.15
PaaG	enoyl-CoA hydratase	0.15
BatB	autotransporter	0.16
ThiC	phosphomethylpyrimidine synthase	0.16
Prn	pertactin autotransporter	0.16

1 Ratio of the amount of whole cell lysate proteins of Δ*bspR* against wild-type *B*. *bronchiseptica*. P-value <0.05.

## Discussion

In this study, we performed comparative profiling of the proteins secreted by *B*. *bronchiseptica* and characterized a hypothetical protein, BB1639, as a novel type III secreted protein, BspR. Alignment of BspR showed different N-terminal sequences among *Bordetella* species (Fig. 1B). The theoretical size of BspR in *B*. *bronchiseptica* was 24.7 kDa (226 amino acids) [3]. By contrast, the theoretical size of BspR was 25.0 kDa (232 amino acids) in *B*. *pertussis* and *B*. *parapertussis*. Unexpectedly, the immunoblot analysis showed that the actual size of BspR was approximately 20 kDa (Fig. 1C). We also confirmed that a 20-kDa BspR species was detected in the whole-cell lysates and supernatants of the *B*. *pertussis* Tohama I strain and several clinical isolates of *B*. *pertussis* (data not shown). For this reason, we analyzed annotated sequence of BB1639 by MetaGeneAnnotator (http://metagene.cb.k.u-tokyo.ac.jp/) that is able to predict the ribosome-binding site of prokaryotic genes [26]. This program assigned the BB1639 open reading frame of 191 amino acids, but not 226 amino acids. Taken together, these results suggest that BspR is probably 20.4 kDa (191 amino acids), with the start codon at amino acid 36 of the originally annotated sequence (denoted by the asterisk in Fig. 1B).

In this study, we complemented the *bspR* mutant strain by the *fhaB* promoter-driven *bspR* expression vector. The *fhaB* promoter-driven vector produces certain amount of BspR that allows effective complementation of the *bspR* mutant. To exclude the unexpected feed back effect on *fhaB* promoter by produced BspR, we also confirmed that Δ*bspR* was complemented by the *bspR* expression vector with its own promoter (Fig. S2).

The lack of BspR resulted in the increased expression of the T3SS, leading to enhancement of T3SS-dependent cytotoxicity and hemolysis in cultured mammalian cells (Fig. 2A, B, and C). Therefore, Δ*bspR* appears to be shifted in the hyper-virulent strain. However, *in vivo* infection demonstrated that the virulence of the Δ*bspR* strain was attenuated relative to that of wild-type strain (Fig. 3D). We reasoned that attenuated virulence of the Δ*bspR* strain can be attributed to dysregulation of virulence factors, such as FhaB and CyaA (Fig. 4). Indeed, β-hemolytic activity observed in the BG agar plate is poorly detected in Δ*bspR* strain and this activity is dependent on the CyaA, but not the T3SS (data not shown). Furthermore, whole-cell proteome analysis clearly indicates that BspR affects expression of various types of bacterial proteins (Table 2, 3, and S1). These findings suggest that the coordinate expression of virulence and non-virulence genes are required for the systemic infection of *B. bronchiseptica*.

The bacterial transcription initiation is regulated by the sigma factor, which associates with the catalytic core RNA polymerase (RNAP) to form the holoenzyme [27], [28]. All bacteria possess at least one essential sigma factor, and alternative sigma factors are involved in the specific gene expression in response to the environmental conditions. In addition, the activity and availability of sigma factors are controlled, in part, by anti-sigma factors that bind and repress their cognate sigma factor. To date, the sigma factor-28 (σ^28^) and anti-sigma factor (FlgM) interaction is the most extensively characterized in several bacteria and both factors are involved in the regulation of the bacteria flagellum assembly [27]. The crystal structure of the σ^28^/FlgM complex revealed that a single FlgM composed of three α-helices wraps around a compact σ^28^ molecule [29]. As a result, FlgM sterically occludes the RNAP-binding determinants of σ^28^ and stabilizes extensive interdomain interactions within σ^28^ that mask the promoter-binding determinants of σ^28^ [29]. The *bspR* is located directly upstream of *btrS*, whose product activates the expression of T3SS genes in the BvgAS-activated phase [23]. BtrS belongs to a family of ECF sigma factors [22], [23], but analysis of deletion mutants of BtrW and BtrV revealed that neither is involved in the expression of T3SS genes, nor do they contribute to the post-transcriptional regulation of the T3SS (i.e., intracellular stability and secretion). Thus, anti-sigma factor for BtrS has not been identified in *Bordetella* species [23] and BspR does not show any sequence similarity to previously described anti sigma factors or regulators. Furthermore, we carried out the GST-pull down assay, but we failed to detect interaction between BspR and BtrS sigma factor (data not shown).

In this study, we demonstrated that BspR functions as a regulator of various genes (Fig. 2, Table 2 and 3). We also found that the *btrS* expression was induced by the Δ*bspR* strain (Fig. 3C) and similar results were obtained by the whole-cell proteome analysis (The Δ*bspR*:wild type ratio = 4.17; Table S1). In addition, the endogenous *bspR* expression was repressed by the introduction of the FLAG-tagged *bspR* expression vector into *B. bronchiseptica* wild type (data not shown), suggesting that BspR negatively regulates its own gene on the *btr* locus.

To our surprise, the relative amount of BvgA in the Δ*bspR* was significantly higher that in *B. bronchiseptica* wild type (Table 2). Although the precise mechanism of the BvgA regulation by BspR remains to be elucidated, BspR may functions as a molecular switch via alteration of the BvgA concentration. Indeed, previous report of BipA in *B. pertussis* indicates that the BvgA concentration has the critical role in the alteration of the Bvg-regulated phases [30]. The *bipA* encodes a large outermembrane protein with similarity to intimin of enteropathogenic *Echerichia coli* and invasin of *Yersinia* spp. [31], and is maximally expressed in the Bvg^i^ phase [30]. The *in vitro* transcription assay clearly demonstrated that BvgA functions as both positive and negative regulators for the *bipA* transcription in the Bvg^i^ phase expression [30]. It has been also reported that the *bipA* promoter region was incubated with RNA polymerase (RNAP) in the various concentrations of BvgA-phosphate (BvgA∼P) *in vitro* and transcriptional activation of *bipA* was induced at concentrations of BvgA∼P between 20 and 220 nM [30]. In contrast, the transcriptional activity was reduced at concentrations of BvgA∼P between 220 and 600 nM. Thus, relatively low concentrations of BvgA∼P are required for activation of *bipA* trancription. Similar results were also obtained by our proteome analysis. The BipA expression was significantly decreased in the *bspR* mutation (Table 3), where the abundance of BvgA was significantly increased. In *Bordetella* species, the BvgAS system functions as a master regulator of the various gene expression icluding *btrS* on the *btr* locus (Table 2). Collectively, these results suggest that BspR regulates *btrS* and other gene expressions via the alteration of the BvgA concentration, rather than the anti sigma factor for BtrS.

Secreted regulators have been reported in some bacterial species. ExsE is a protein secreted via the T3SS in *Pseudomonas aeruginosa* and has the ability to bind to an anti-anti-activator, ExsC, before secretion [32], [33]. The decrease in intracellular ExsE levels caused by its secretion results in the release of ExsC. The released form of ExsC associates with ExsD, resulting in the disruption of the ExsD-ExsA complex. Finally, liberating the transcriptional activator ExsA promotes the transcription of the *Pseudomonas* T3SS operon. Vp1702 in *Vibrio parahaemolyticus* has no sequence similarity with ExsE, but deletion of Vp1702 results in the enhanced secretion of type III-associated proteins, and Vp1702 itself is secreted via the T3SS in *V*. *parahaemolyticus* [34]. Thus, the secretion of a negative regulator via the T3SS may be a common strategy for the regulation of T3SSs among various bacterial species. One striking feature of BspR is its role as a secreted regulator of iron-responsive virulence genes.

The transcription of most iron-responsive genes is controlled by the ferric uptake regulator (Fur) protein [35]. Under iron-replete conditions, the iron-bound form of Fur directly binds to the consensus sequence in the upstream of target genes (e.g., those encoding iron acquisition systems) to repress their transcription [36]. Although the *Bordetella* T3SS is also controlled by the iron concentration, a typical Fur binding site has not been found in the intergenic region upstream of the *bsc* or *btr* locus [25]. In this study, we found that the phenotype of iron-starved wild-type *B*. *bronchiseptica*, including the hyper-secretion of type III-associated proteins, was similar to that of the Δ*bspR* strain. Furthermore, the secretion of type III-associated proteins decreased in response to an increase in the iron concentration, but this stringent regulation was relieved in the absence of BspR. Thus, BspR is involved in the iron-responsive gene expression of virulence factors.

The cross-talk between the T3SS and flagella has been reported in several gram-negative bacteria. Over-production of ExsA results in the reduction of flagellar gene expression and motility in *P*.* aeruginosa* [37]. Conversely, both a *P*. *aeruginosa* mutant lacking the flagellar filament and a mutant deficient for GacA, which is a positive regulator of motility, show hyper-secretion of ExoS, a T3SS effector [37]. In *Bordetella*, the motility and synthesis of flagella are negatively regulated by the BvgAS system [38], indicating that the T3SS and flagella are inversely regulated in *Bordetella* species. In this study, we speculate that BspR may function as a molecular switch by alteration of the BvgA concentration and the absence of iron is an environmental cue for the host internalization. Our data suggest that virulence factors are coordinately and inversely regulated by BspR, depending on the bacterial infection stage. The precise molecular mechanism of the effect of BspR on virulence gene expression under iron starvation or iron-rich conditions remains to be elucidated. The model of BspR-mediated gene regulation provides new insight into how *Bordetella* species establish the virulent phase in response to environmental signals.

## Materials and Methods

### Ethics Statement

This study was carried out in strict accordance with the recommendations in the Guide for the Care and Use of Laboratory Animals of the Ministry of Education, Culture, Sports, Science and Technology of Japan and Social Council of Japan. The protocol was approved by the Committee on the Ethics of Animal Experiments of Kitasato University (Permit Number: 11014). All surgery was performed under sodium pentobarbital anesthesia, and all efforts were made to minimize suffering.

### Bacterial Strains, Cell Culture and Growth Media

The wild-type strain used in this study was *Bordetella bronchiseptica* S798 [15]. The isogenic type III secretion mutant (ΔT3SS), which is deficient in *bscN* ATPase for the type III secretion, was derived from S798 [15]. *Bordetella* strains were cultured in Stainer and Scholte (SS) medium with a starting *A*_600_ of 0.2 under vigorous shaking at 37°C, and the inoculum was prepared from fresh colonies grown on Bordet and Gengou (BG) agar as described previously [39], [40], [41]. The wild-type *B*. *bronchiseptica* grown in SS medium containing MgSO_4_ at a final concentration of 40 mM was used as a negative control in this study because of the inactivation of BvgAS-regulated virulence genes [9]. The liquid cultivation period was 18 h for the protein preparation experiments and infection assays and 9 h for mRNA preparation. L2 (ATCC CCL-149) and HeLa (ATCC CCL-2) cells were maintained in F-12K (Invitrogen) and Eagle’s minimum essential medium (EMEM) (Sigma), respectively, each containing 10% fetal calf serum at 37°C under an atmosphere of 5% CO_2_.

### Proteomic Analysis Using iTRAQ Reagents and Tandem Mass Spectroscopy

Bacteria were cultured in SS medium at 37°C under vigorous shaking for 18 h as described above. To prepare the total secreted protein samples, the culture supernatants of wild-type and ΔT3SS *B. bronchiseptica* were centrifuged followed by filtration. The clarified supernatants were concentrated by ultrafiltration using Centriprep columns with a 10,000 nominal molecular weight limit (Millipore) and were reconstituted with PBS. The proteins were denatured with SDS, reduced with Tris(2-carboxyethyl)phosphine (TCEP), alkylated with methyl methanethiosulfonate (MMTS), and then digested with trypsin (Applied Biosystems) at 37°C for 18 h. The resulting peptides derived from the proteins secreted by wild-type and ΔT3SS *B. bronchiseptica* were labeled with the 116 and 119 iTRAQ tags (Applied Biosystems), respectively. The labeled peptides were mixed, followed by fractionation using strong cation exchange (SCX) chromatography (Cation Exchange Buffer Pack, Applied Biosystems). Each fraction was subjected to mass spectrometry analysis using a QSTAR Elite Hybrid LC/MS/MS System (Applied Biosystems) and a DiNa System (KYA TECH Corp.). The MS-MS spectrum data were processed using Protein Pilot™ Software 2.0.1 (Applied Biosystems), with the Paragon algorithm [42] by comparison with the genomic database for *B*. *bronchiseptica* RB50. Protein identification was performed with a confidence threshold of a Protein Pilot™ Unused Score >2.0. The heights of the iTRAQ reporter ion peak in the MS/MS spectra were also calculated by Protein Pilot™ to determine the ratio relative to the reporter. For the analysis of the protein profile of whole-cell lysate, wild-type or Δ*bspR B. bronchiseptica* was cultured in SS medium at 37°C under vigorous shaking for 18 h as described above. The bacterial culture was centrifuged to be precipitated and resuspended with B-PER (Piece) to be lysed. The crude bacterial lysate was clarified by centrifugation and was processed to be trypsinized as described above. The resulting peptides derived from the whole-cell lysates of wild-type and Δ*bspR B. bronchiseptica* were labeled with the 114 and 117 iTRAQ tags (Applied Biosystems), respectively. The labeled peptides were subjected to the mass spectrometry analysis. As described above, the resulting MS/MS spectra was processed by comparison with the genomic database for *B*. *bronchiseptica* RB50, *B*. *pertussis* Tohama I, and *B*. *parapertussis* 12822 for the protein identifications.

### Primers

The primers used in this study are listed in Table 4.

**Table 4:** Primers used in this study.

Primer	Sequence
B1-*bspR*	5′-AAAAAGCAGGCTCGAAATGTTCGCTGCGCGCG-3′
B2-*bspR*	5′-AGAAAGCTGGGTCGCGCGCAGCGAACATTTCG-3′
R1-*bspR*	5′-CGCGGATCCCATGGAATATGGAATATGCA-3′
R2-*bspR*	5′-CGCGGATCCCATACGGCGCAAGAATGGCT-3′
B1-*bspR* comp	5′-AAAAAGCAGGCTACTCTCCGCGTTGACGGCGC-3′
B2-*bspR* comp	5′-AGAAAGCTGGGTCTACATCAGGTGGTGCGCAA-3′
5-*bspR*-pCX340-NdeI	5′-GGAATTCCATATGACTCTCCGCGTTGACGGCGC-3′
3-*bspR*-pCX340-KpnI	5′-GGGGTACCCAGGTGGTGCGCAAGGACCT-3′
5-recA	5′-CGCTGGACGTGCAATAC-3′
3-recA	5′-CATCTCGCCTTCGATTTC-3′
5-bsp22	5′-ATGGTGTATGTGCAGGG-3′
3-bsp22	5′-TTCGGATTGGGCGGAAAC-3′
5-bopN	5′-GGCTTCCAGCACGCGTA-3′
3-bopN	5′-CATAGCGTTCCAGCACC-3′
5-bteA	5′-CAGCACCGATTTCGAGG-3′
3-bteA	5′-CGCTCATCTCGATGTTGG-3′
5-btrS	5′-TATCAATCGTTTCGTGGCG-3′
3-btrS	5′-GCGACAAGTGATTGCGG-3′
5-fhaB	5′-CATATCATCAACAGCGCCAA-3′
3-fhaB	5′-GAAATCCTCCACATCCAGCA-3′
5-prn	5′-CATGACGACCAGCTTGT-3′
3-prn	5′-GTCCTGATGGTCGATACG-3′

* The underlined portions (GGATCC) indicate the BamHI site for the self-ligation of the inverse PCR products in the construction of mutants.

### Construction of the *bspR* Mutant

pDONR221 (Invitrogen) and pABB-CRS2 [43] were used as cloning and positive suicide vectors, respectively. A 2.6-kbp DNA fragment containing the *bspR* gene and its flanking region was amplified by PCR with the primers B1-*bspR* and B2-*bspR* using *B. bronchiseptica* S798 genomic DNA as the template. The resulting PCR product was cloned into pDONR221 using the adaptor PCR method (Gateway cloning system, Invitrogen) to obtain pDONR-*bspR*. Then, for the deletion of *bspR* in pDONR-*bspR*, inverse PCR was carried out with the primers R1-*bspR* and R2-*bspR* using circular pDONR-*bspR* as a template. The resulting PCR products were digested with BamHI and self-ligated to obtain pDONR-Δ*bspR*, which contained a BamHI site in addition to a 579-bp deletion including the start codon of *bspR*. pDONR-Δ*bspR* was ligated with pABB-CRS2 to obtain pABB-CRS2-Δ*bspR* using the Gateway cloning system. pABB-CRS2-Δ*bspR* was then introduced into *E. coli* SM10λ*pir* and was transconjugated into *B. bronchiseptica* S798, as described previously [44]. The resulting mutant strain was designated Δ*bspR*.

### Construction of Plasmids for Bacterial Gene Expression

For the complementation of the *bspR* defect in Δ*bspR*/p*bspR* was constructed as follows [18]. An 881-bp fragment encoding *bspR* was amplified by PCR with B1-*bspR* comp and B2-*bspR* comp primers using S798 genomic DNA as a template. The resulting fragment was cloned into pDONR221, and this construct was designated pDONR-*bspR*-comp. To express BspR in *B. bronchiseptica*, pDONR-*fha*P (*fha* promoter), pDONR-*bspR*-comp, and pDONR-*rrnB* (*rrnB* terminator) were mixed and cloned into the pRK415 R4-R3-F vector using the MultiSite Gateway system (Invitrogen) to obtain p*bspR*. For the construction of the *bspR* expression vector under the control of its own promoter, pDONR-*bspR*-comp was cloned into pABB415 vector ([17]) to obtain pABB415-*bspR*.

### Immunoblot Analysis

The secreted proteins released into bacterial culture supernatants and bacterial whole-cell lysates were isolated by trichloroacetic acid precipitation. The culture supernatants were filtered, and the bacterial pellets were resuspended in distilled water. Trichloroacetic acid was then added to the samples at a final concentration of 10%. After incubation of the samples on ice for 15 min, they were centrifuged for 5 min. The resulting precipitated proteins were neutralized with 2 M Tris-base and dissolved in sample buffer. The protein samples were separated by SDS-PAGE and analyzed by CBB staining or immunoblot analysis.

### Antibodies

To prepare the anti-BspR antibodies, peptides corresponding to the amino acid residues 136 to 153 of BspR (CHDTGVSPDQISGTLRAT) were cross-linked with a carrier protein by the cysteine residue at the N-terminus. The cross-linked peptides were used to immunize rabbits. To obtain specific immunoglobulin fractions, all antisera obtained were adsorbed to the peptide immobilized on sepharose. The anti-BteA, anti-BopB, anti-BopD, anti-Bsp22, anti-filamentous hemagglutinin (FhaB) and anti-pertactin (Prn) antibodies used in this study have been described previously [15], [17], [18], [45]. The anti-adenylate cyclase toxin (CyaA) antibodies were purchased from Santa Cruz Biotechnology. The anti-TEM-1 β-lactamase antibodies were purchased from QED bioscience Inc. The anti-dermonecrotic toxin (DNT) antibodies were a gift from Yasuhiko Horiguchi.

### mRNA Analysis by RT-PCR

The mRNA levels were measured by quantitative reverse-transcription PCR (qRT-PCR). Bacterial total RNA was isolated with the RNeasy Mini Kit (QIAGEN), and the RNA sample was reverse-transcribed with Omniscript Reverse Transcriptase (QIAGEN) with random primers. The resulting cDNA was amplified with SYBR Premix Ex Taq (TAKARA) with the following primer pairs: 5-recA and 3-recA for *recA*, 5-bsp22 and 3-bsp22 for *bsp22*, 5-bopN and 3-bopN for *bopN*, 5-bteA and 3-bteA for *bteA*, 5-btrS and 3-btrS for *btrS*, 5-fhaB and 3-fhaB for *fhaB*, and 5-prn and 3-prn for *prn*. The expression of *recA* was used as an internal control. The specificity of each primer set was checked by analyzing the melting curves, and results were calculated using the comparative cycle threshold method, in which the amount of *bsp22*, *bopN*, *bteA*, *btrS*, *fhaB*, or *prn* mRNA is normalized to the amount of *recA* mRNA and reported as arbitrary units set to a value of 1 for the wild-type strain.

### Infection Assays

Morphological changes in infected cells was observed as previously described [15], except that cells were infected at an multiplicity of infection (MOI) of 20. L2 (ATCC CCL-149) cells (1×10^5^) seeded on coverslips in 6-well plates were infected with bacteria at an MOI of 20. The cells were then centrifuged for 5 min and incubated for 20 min at 37°C in an atmosphere of 5% CO_2_. The cells were washed with phosphate-buffered saline (PBS) and fixed in methanol. Fixed cells were stained with Giemsa solution (Merck) and analyzed by microscopy (Axioplan 2 Imaging, Zeiss). To examine the release of lactate dehydrogenase (LDH) from infected cells, 7.5×10^4^ HeLa (ATCC CCL-2) cells seeded in 24-well plates were infected with bacteria at an MOI of 10. The cells were then centrifuged for 5 min and incubated at 37°C in an atmosphere of 5% CO_2_ for the indicated amount of time. The amount of LDH was measured spectrophotometrically using a Cyto-Tox 96 non-radioactive cytotoxicity assay kit (Promega). The relative amount of LDH release (%) was calculated as follows: experimental LDH activity/total LDH activity×100. The total LDH activity was measured using cells treated with 1% Triton X-100.

### Hemolysis Assay

The measurement of type III-dependent hemolytic activity was carried out as described previously [15]. Briefly, bacterial pellets from overnight cultures and rabbit red blood cells (RBCs) were washed with PBS and adjusted to 5×10^10^ bacteria/ml and 3×10^9^ cells/ml, respectively. The suspensions were mixed together (using 50-µl aliquots per suspension) in 96-well plates and centrifuged for 5 min to promote close contact. The combined suspensions were then incubated at 37°C for 30 min in a CO_2_ incubator. An additional 100 µl of PBS was added to the mixtures of bacteria and RBCs, and then the plates were centrifuged again. The supernatants were transferred to new plates, and the optical density was measured at 492 nm. In a previous study, we confirmed that the hemolytic activity induced by the adenylate cyclase toxin can be excluded using this measurement system [15].

### *In vivo* Infection of Mice

C57BL/6J mice (5 to 6 weeks old) were purchased from CLEA Japan, Inc. After arrival, all mice were housed for 1 week before experiments. Mice were infected intranasally with 50 µl (5×10^6^ CFU) of a *B*. *bronchiseptica* suspension prepared from an individually isolated colony on BG agar plates.

### Statistical Analysis

Survival curves were generated by the Kaplan-Meier method, and statistical analyses were performed using the log-rank test. The statistical significance was assessed by Student’s t-tests. A P-value, <0.05 was considered significant.

## Supporting Information

Figure S1 **Secreted proteins isolated from** ***B*** **.** ***bronchiseptica*** **culture supernatants.** Wild-type and ΔT3SS *B. bronchiseptica* strains were grown in SS medium under vigorous shaking at 37°C for 18 h. The secreted proteins isolated from bacterial culture supernatants were separated by SDS-PAGE and stained with CBB.
(TIF)

Figure S2 **Complementation of the** Δ***bspR*** **strain.** The *fhaB* promoter-driven expression vector of *bspR*, p*bspR* (lane 3), *bspR* expression vector by its own promoter, pABB415-*bspR* (lane 4), or the empty vector of pRK415 (lane 5) was introduced into the Δ*bspR* strain, respectively. *Bordetella bronchiseptica* wild type (lane 1), Δ*bspR* (lane 2), and Δ*bspR* complemented strains (lanes 3 and 4) were grown in SS medium under vigorous shaking at 37°C for 18 h. The secreted proteins isolated from bacterial culture supernatants were separated by SDS-PAGE and stained with CBB (lower panel). The whole-cell lysates were analyzed by immunoblotting using anti-BspR antibodies (upper panel).
(TIF)

Table S1 (XLS)
