# Supplementary figures and images for: The Type III Secreted Protein BspR Regulates the Virulence Genes in Bordetella bronchiseptica
Source: PLoS One. 2012 Jun 11;7(6):e38925. doi: 10.1371/journal.pone.0038925 (PMC3372540; doi:10.1371/journal.pone.0038925)

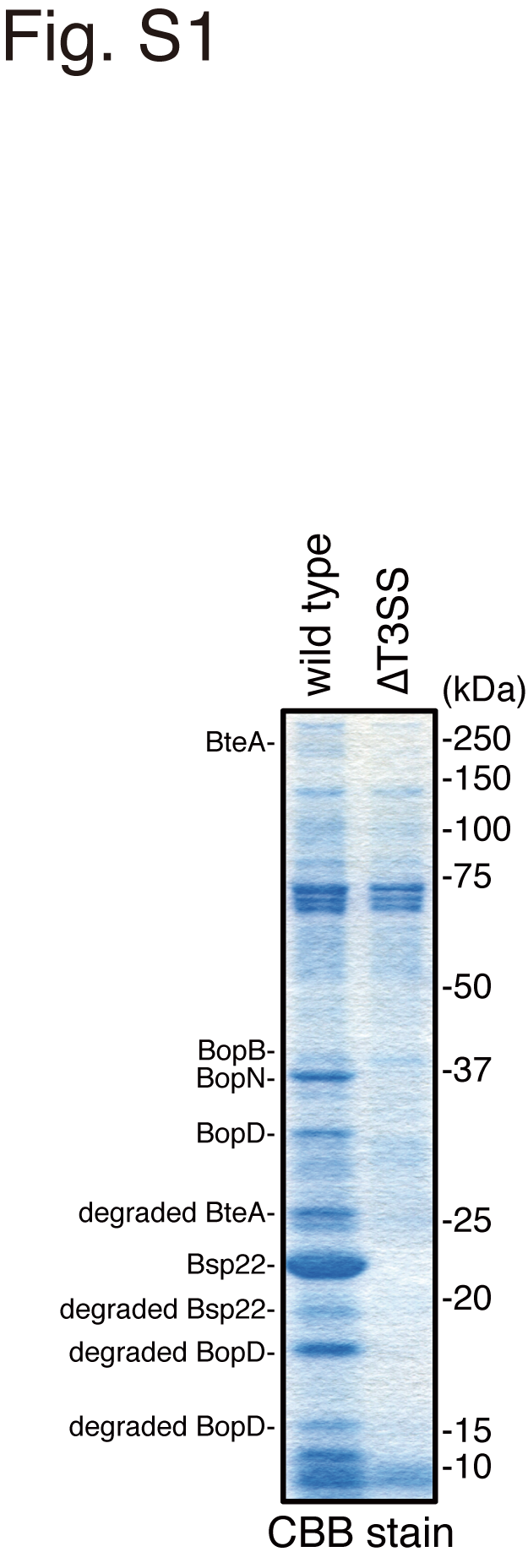

Supplement: Figure S1 — Secreted proteins isolated from B . bronchiseptica culture supernatants. Wild-type and ΔT3SS B. bronchiseptica strains were grown in SS medium under vigorous shaking at 37°C for 18 h. The secreted proteins isolated from bacterial culture supernatants were separated by SDS-PAGE and stained with CBB. (TIF) [file pone.0038925.s001.tif]

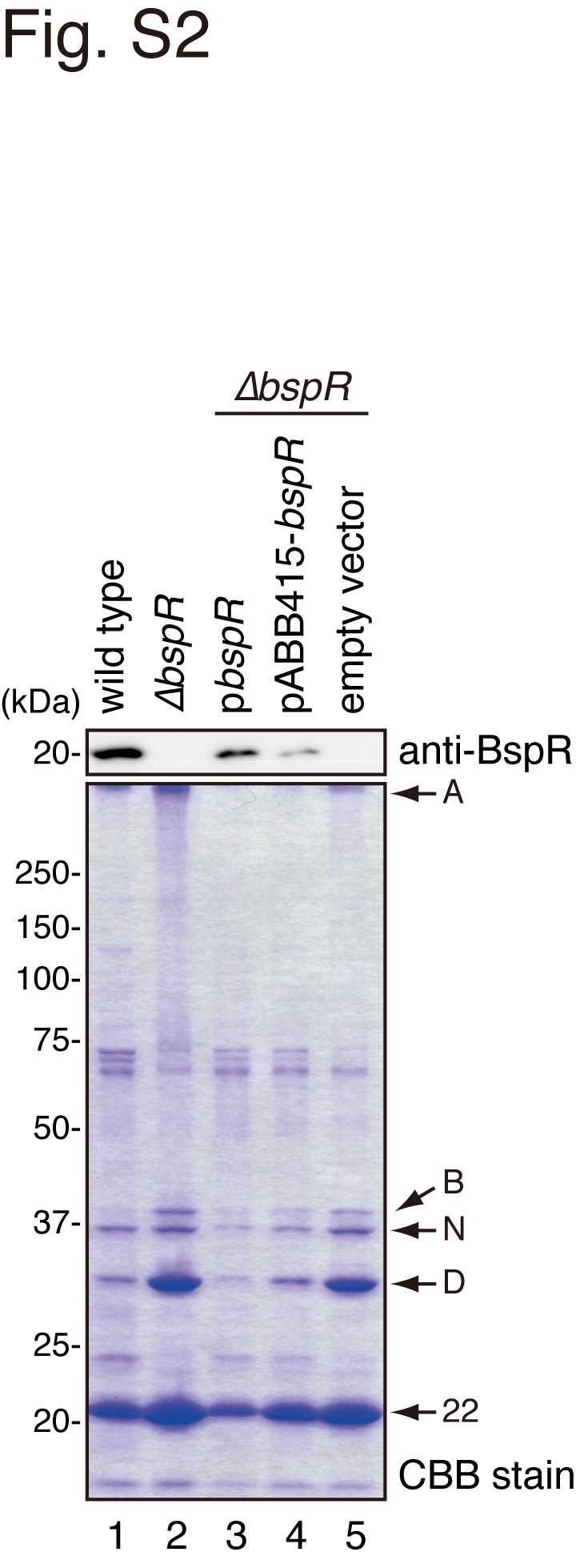

Supplement: Figure S2 — Complementation of the ΔbspR strain. The fhaB promoter-driven expression vector of bspR, pbspR (lane 3), bspR expression vector by its own promoter, pABB415-bspR (lane 4), or the empty vector of pRK415 (lane 5) was introduced into the ΔbspR strain, respectively. Bordetella bronchiseptica wild type (lane 1), ΔbspR (lane 2), and ΔbspR complemented strains (lanes 3 and 4) were grown in SS medium under vigorous shaking at 37°C for 18 h. The secreted proteins isolated from bacterial culture supernatants were separated by SDS-PAGE and stained with CBB (lower panel). The whole-cell lysates were analyzed by immunoblotting using anti-BspR antibodies (upper panel). (TIF) [file pone.0038925.s002.tif]
